# Combinatorial gene targeting in primary human hematopoietic stem and progenitor cells

**DOI:** 10.1038/s41598-022-23118-8

**Published:** 2022-10-28

**Authors:** Alexandra Bäckström, David Yudovich, Kristijonas Žemaitis, Ludvig Nilsén Falck, Agatheeswaran Subramaniam, Jonas Larsson

**Affiliations:** Division of Molecular Medicine and Gene Therapy, Lund Stem Cell Center, Lund University, BMC A12, 221 84 Lund, Sweden

**Keywords:** Haematopoietic stem cells, Genetics, Molecular biology

## Abstract

The CRISPR/Cas9 system offers enormous versatility for functional genomics but many applications have proven to be challenging in primary human cells compared to cell lines or mouse cells. Here, to establish a paradigm for multiplexed gene editing in primary human cord blood-derived hematopoietic stem and progenitor cells (HSPCs), we used co-delivery of lentiviral sgRNA vectors expressing either Enhanced Green Fluorescent Protein (EGFP) or Kusabira Orange (KuO), together with Cas9 mRNA, to simultaneously edit two genetic loci. The fluorescent markers allow for tracking of either single- or double-edited cells, and we could achieve robust double knockout of the cell surface molecules CD45 and CD44 with an efficiency of ~ 70%. As a functional proof of concept, we demonstrate that this system can be used to model gene dependencies for cell survival, by simultaneously targeting the cohesin genes STAG1 and STAG2. Moreover, we show combinatorial effects with potential synergy for HSPC expansion by targeting the Aryl Hydrocarbon Receptor (AHR) in conjunction with members of the CoREST complex. Taken together, our traceable multiplexed CRISPR/Cas9 system enables studies of genetic dependencies and cooperation in primary HSPCs, and has important implications for modelling polygenic diseases, as well as investigation of the underlying mechanisms of gene interactions.

## Introduction

Hematopoietic stem cells (HSCs) are tightly regulated by both extrinsic and intrinsic regulators such as signals from the bone marrow microenvironment, complex transcription factor networks and epigenetic regulators^1–3^. HSCs are unique among somatic stem cells due to their accessibility and the possibility to perform precise functional modelling both in vitro and in vivo. To study gene function in human HSCs, viral vectors for gene overexpression or knockdown by RNA interference (RNAi) have been widely used and greatly contributed to the understandings of HSC biology^4–6^. The CRISPR/Cas9 system for gene editing has greatly expanded the molecular toolbox and been widely applied to genetically modify or transcriptionally regulate specific genetic loci. For many applications, such as modelling of complex diseases or functional studies of gene interactions, combinatorial genetic perturbations are desired^7^. Even though the CRISPR/Cas9 system allows for multiplexed gene editing, this has mainly been applied in immortalized human cell lines and mouse cells where standard CRISPR/Cas9 delivery vectors are well tolerated^8,9^. In sensitive primary human cell types, such as hematopoietic stem and progenitor cells (HSPCs), the CRISPR/Cas9 components are commonly delivered as ribonucleoproteins (RNPs), providing better efficiency and tolerability^10–12^. Although several CRISPR/Cas9 RNP complexes can be used to edit more than one locus in parallel^13^, this method does not allow for tracing of edited cells to discriminate them from non-edited cells.

We have previously reported on a split CRISPR/Cas9 delivery system, combining stable lentiviral sgRNA delivery and transient delivery of Cas9 mRNA, for efficient and traceable gene editing in primary CD34^+^ HSPCs^14^. The use of lentiviral sgRNA vectors containing fluorescent markers allows extension of this system to traceable multiplexed perturbations^15^. Here, we have therefore optimized the approach for efficient editing of multiple genetic loci in cord blood-derived CD34^+^ cells, and show efficient double knockout of the cell surface markers CD45 and CD44. Further, as proof of concept, we demonstrate the feasibility of the system to study both genetic dependencies and co-operative actions between genes in primary human HSPCs.

## Results

### Efficient and traceable double gene editing in human HSPCs

Here, we set out to explore different strategies to simultaneously knockout two genes using our recently published split CRISPR/Cas9 delivery system in cord blood (CB)-derived CD34^+^ HSPCs^14^. In our previous work, using a chimeric sgRNA backbone, we identified highly efficient sgRNAs targeting the two cell surface markers CD45 and CD44. These markers are abundantly expressed in HSPCs, allowing for easy detection of successful editing by flow cytometry^14^. We first assessed double editing of CD45 and CD44 by co-expressing the two sgRNAs from a single lentiviral vector. We therefore added an additional pol III promoter, H1^16^, to our previously established U6 driven sgRNA vector^14^ to drive the expression of a second sgRNA (Supplementary Fig. S1). In contrast to the high editing efficiency that we had previously achieved using single sgRNA vectors, we found that combining two promoter-sgRNA cassettes in one vector resulted in very low editing efficiency, with less than 5% of the transduced cells showing double knockout of CD45 and CD44 following Cas9 mRNA delivery (Supplementary Fig.S1). This could possibly be due to the H1 and U6 promoters interfering with each other, and we therefore looked for alternative strategies to express the two sgRNAs. Previously, the use of small tRNA promoters to express sgRNAs in tandem has been described^17,18^. From a single transcript, several sgRNAs can be produced using the endogenous tRNA-processing system. To test this approach in CD34^+^ HSPCs, we constructed vectors containing the U6 promoter followed by two tRNA-gRNA units (Supplementary Fig. S1). Using these vectors, we were able to achieve better editing in both genes reaching 16% CD45^−^CD44^−^ cells (Supplementary Fig. S1). Yet, a majority of the transduced cells were not edited, which would limit subsequent functional experiments in HSPCs using this system.

As an alternative approach, we delivered Cas9:sgRNA RNPs according to standard protocols to simultaneously edit CD45 and CD44. However, similar to the bicistronic vectors, this approach created a mixed population of non-edited, single-edited and double-edited cells with the majority of the cells remaining non-edited (Supplementary Figure S1).

**Figure 1:**
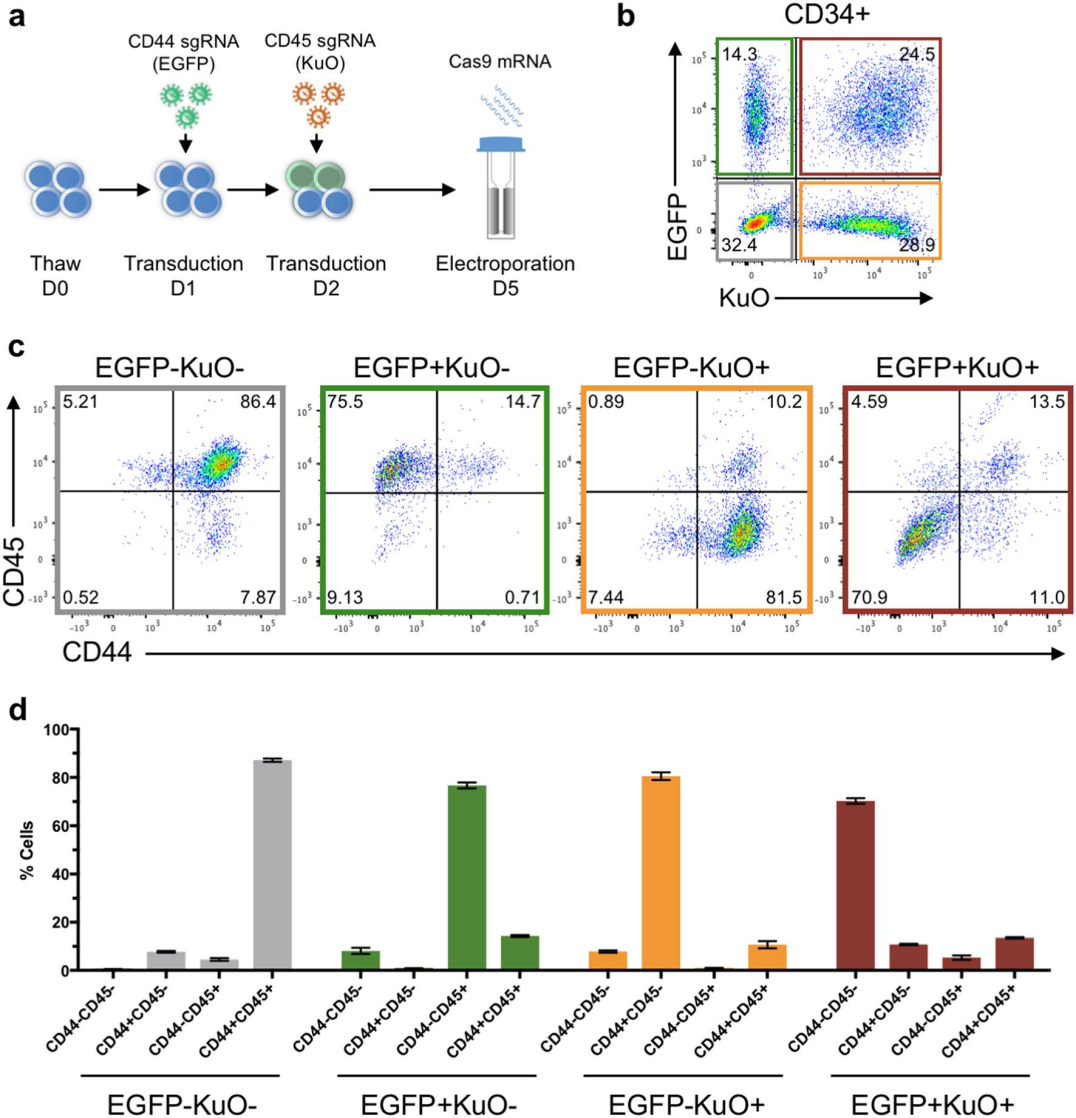
Efficient and traceable double gene editing of CD44 and CD45 in primary CD34^+^ HSPCs. (**a**) Schematic overview of experimental outline. Thawed CD34^+^ cells were sequentially transduced with lentiviral sgRNA targeting CD44, containing EGFP, on day 1 followed by transduction with lentiviral sgRNA targeting CD45, containing KuO, on day 2. Cells were electroporated with Cas9 mRNA on day 5 (n = 3). (**b**) Representative FACS plot of the transduction efficiency of EGFP- and KuO-containing sgRNA vectors in CD34^+^ cells 4 days post-electroporation. (**c**) Representative FACS plots of CD44 and CD45 editing in the transduced populations marked in (**b**). (**d**) Frequency of cells expressing CD45 and/or CD44 in the transduced populations marked in (**b**).

Given the modest double knockout efficiencies we had observed using both bicistronic sgRNA vectors and RNP delivery, we next sought to express the two sgRNAs from separate vectors with different fluorescent markers. This approach would further enable subsequent tracing of cells having received one of the two vectors, or both. We created a vector containing Kusabira Orange (KuO) and the sgRNA targeting CD45 to be used in combination with our previously established Enhanced Green Fluorescent Protein (EGFP)-containing vector with the CD44 sgRNA. We noted that sequential transduction, delivering the first sgRNA vector on day 1 followed by the second sgRNA vector on day 2, rendered substantially higher levels of double transduced cells compared to co-transductions on the same day (Supplementary Fig. S1). CD34^+^ HSPCs is a heterogeneous cell population with different transduction propensities^19,20^. We observed that the double transduction efficiency in sub fractions of this population differed, with CD34mid and CD34low cells displaying similar frequencies of double transduced cells while the more immature CD34high cells were double transduced to a lesser extent (Supplementary Fig. S1). Following sequential transduction of CD34^+^ cells and subsequent electroporation with Cas9 mRNA, we found that a substantial fraction (around 70%) of the double transduced population (EGFP^+^KuO^+^) had been double-edited and lost expression of both CD45 and CD44 (Fig. 1a–d). Also, the single transduced populations, EGFP^+^KuO^−^ and EGFP^-^KuO^+^, displayed high levels of editing in CD44 and CD45, respectively (Fig. 1c,d).

Taken together, we demonstrate that our split CRISPR/Cas9 delivery system can be used for efficient multiplexed editing in CB-derived CD34^+^ HSPCs. Importantly, the use of separate fluorescent markers for each lentiviral sgRNA vector enables tracing of both single-edited and double-edited populations within the same well of cells.

### Modelling synthetic lethality between the cohesin genes STAG1 and STAG2

**Figure 2:**
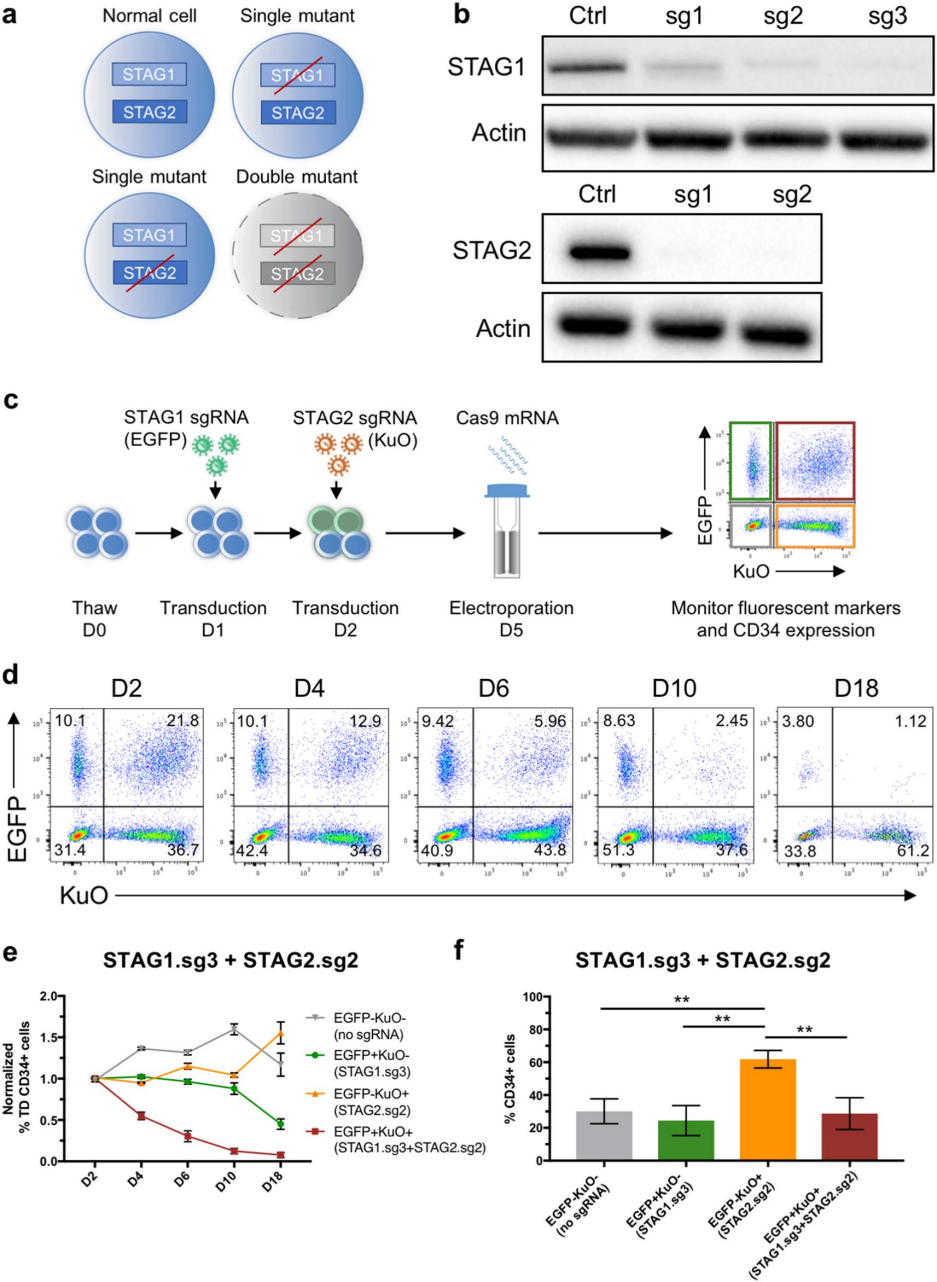
Double gene editing to model the synthetic lethality of STAG1 and STAG2 in primary CD34^+^ HSPCs. (**a**) Illustration of the synthetic lethality concept of STAG1 and STAG2. Disruption in one of the two genes is tolerated while disruption of both genes is lethal to the cell. (**b**) Western blot analysis of STAG1 and STAG2 proteins in untreated Cas9-expressing HL60 cells (Ctrl) and Cas9-expressing HL60 cells 4 days post-transduction with lentiviral sgRNAs targeting STAG1(sg1, sg2, sg3) or STAG2 (sg1, sg2). STAG1 and actin bands, and STAG2 and actin bands, were cropped from different parts of the same blot, respectively. Original blots are shown in Supplementary Figure S4. (**c**) Overview of experimental outline to target STAG1 and STAG2 in primary HSPCs. Thawed CD34^+^ cells were sequentially transduced on day 1 and day 2 with lentiviral sgRNAs targeting STAG1 and STAG2, containing EGFP or KuO, respectively. Cells were electroporated with Cas9 mRNA after 3 days in culture and flow cytometry was used to analyse fluorescent marker and CD34 expression (n = 3). (**d**) Representative FACS plots of the transduction efficiency in CD34^+^ cells for sgRNA combination STAG1.sg3 (EGFP) and STAG2.sg2 (KuO) at day 2–18 (D2–18) post-electroporation. (**e**) Normalized frequency of transduced (TD) CD34^+^ cells at day 2–18 (D2–18) post-electroporation for sgRNA combination STAG1.sg3 and STAG2.sg2. (**f**) Frequency of CD34^+^ cells in transduced populations at day 18 post-electroporation for sgRNA combination STAG1.sg3 and STAG2.sg2. Statistical significance was calculated using One-Way ANOVA with Tukey’s multiple comparisons test. **p < 0.01.

Having established a paradigm for efficient double gene knockout in CB-derived CD34^+^ HSPCs, we next set out to exploit this in functional contexts by assessing both dependencies and cooperation between genes. Genetic dependencies may occur among genes that have similar functions and that can compensate for the loss of one another^21^. Such gene redundancies can sometimes be associated with synthetic vulnerability, where a perturbation in one of two redundant genes will have a viable outcome but perturbation of both genes will result in cell death^22^. One paralog gene pair that has been demonstrated to have such a synthetic lethal interaction consists of STAG1 and STAG2 (Fig. 2a)^23^. STAG1 and STAG2 are both members of the cohesin complex, and STAG2 is frequently mutated in a variety of human cancers including Ewing’s Sarcoma^24^, bladder cancer^25^, and myeloid neoplasms^26,27^. The synthetic lethal interaction between STAG1 and STAG2 holds great potential for the development of selective therapeutics by targeting STAG1 to selectively eliminate STAG2 mutated cancers^28^.

As a proof of concept, we decided to target STAG1 and STAG2 using our double lentiviral sgRNA vector approach to model potential synthetic lethal interactions in CB-derived CD34^+^ HSPCs. First, we transduced Cas9-expressing HL60 cells with individual lentiviral sgRNA vectors targeting STAG1 or STAG2 to identify efficient sgRNAs using Western blotting. Indeed, 2 out of 3 sgRNAs targeting STAG1 and both sgRNAs targeting STAG2 showed a near complete loss of protein (Fig. 2b). Next, we transduced CD34^+^ cells with lentiviral sgRNA vectors targeting STAG1 (sg3) or STAG2 (sg2), containing EGFP or KuO, respectively, followed by Cas9 mRNA electroporation (Fig. 2c). The expression of EGFP and KuO in the CD34^+^ population was monitored using flow cytometry at day 2, 4, 6, 10 and 18 post-electroporation. As expected, the double transduced EGFP^+^KuO^+^ population declined rapidly between day 2 and day 10 post-electroporation, while the single transduced populations showed stable EGFP or KuO levels, indicating a synthetic lethal effect in the double transduced population (Fig. 2d,e). Moreover, we did not observe such a drastic drop of the double transduced population in control experiments where we targeted the two cell surface markers CD44 and CD45 or when using non-targeting guides (Supplementary Fig. S2).

Knockdown of STAG2 in umbilical CB-derived CD34^+^ HSPCs using shRNA has previously been shown to induce expansion of cells with an immature phenotype *in vitro*^29^. In line with these findings, we saw that the STAG2 targeted population (EGFP^−^KuO^+^) had increased substantially by day 18 and displayed a significantly higher proportion of CD34^+^ cells compared to the other edited cell populations (EGFP^+^KuO^−^ and EGFP^+^KuO^+^), as well as the non-edited (EGFP^−^KuO^−^) population (Fig. 2d–f). Further, Western blotting of sorted KuO^+^ cells harvested on day 13 post-electroporation revealed a near complete loss of STAG2 protein, confirming successful knockout of STAG2 expression in these cells and a functional selection of knockout cells within this population (Supplementary Fig. S2).

In summary, these experiments demonstrate the feasibility of our combinatorial CRISPR/Cas9 gene editing system to model synthetic lethal interactions between genes, and for tracing distinct functional outcomes within the same pool of cells.

### Combinatorial gene targeting of CoREST and AHR to propagate HSPCs ex vivo

Next, to explore the use of our system to study potential combinatorial and synergistic effects of gene perturbations, we targeted Aryl Hydrocarbon Receptor (AHR) as well as two components of the CoREST complex: Lysine (K)-specific Histone Demethylase 1A (LSD1, also known as KDM1A) and REST Corepressor 1 (RCOR1). These gene targets have been identified as ex vivo regulators of hematopoiesis, and perturbing them genetically or by using small molecules has been shown to promote HSPC expansion^30–32^, which is of great relevance in clinical settings of stem cell transplantation using cord blood. Currently, the small molecules StemRegenin 1 (SR1), which is an antagonist of AHR^31^, and UM171, which targets the LSD1-containing CoREST complex^32,33^, are being evaluated in clinical trials^34–36^. While SR1 and UM171 have been used together to promote HSPC expansion, combined genetic targeting of AHR and CoREST has not been studied, and we sought to employ our double knockout system for this purpose.

**Figure 3:**
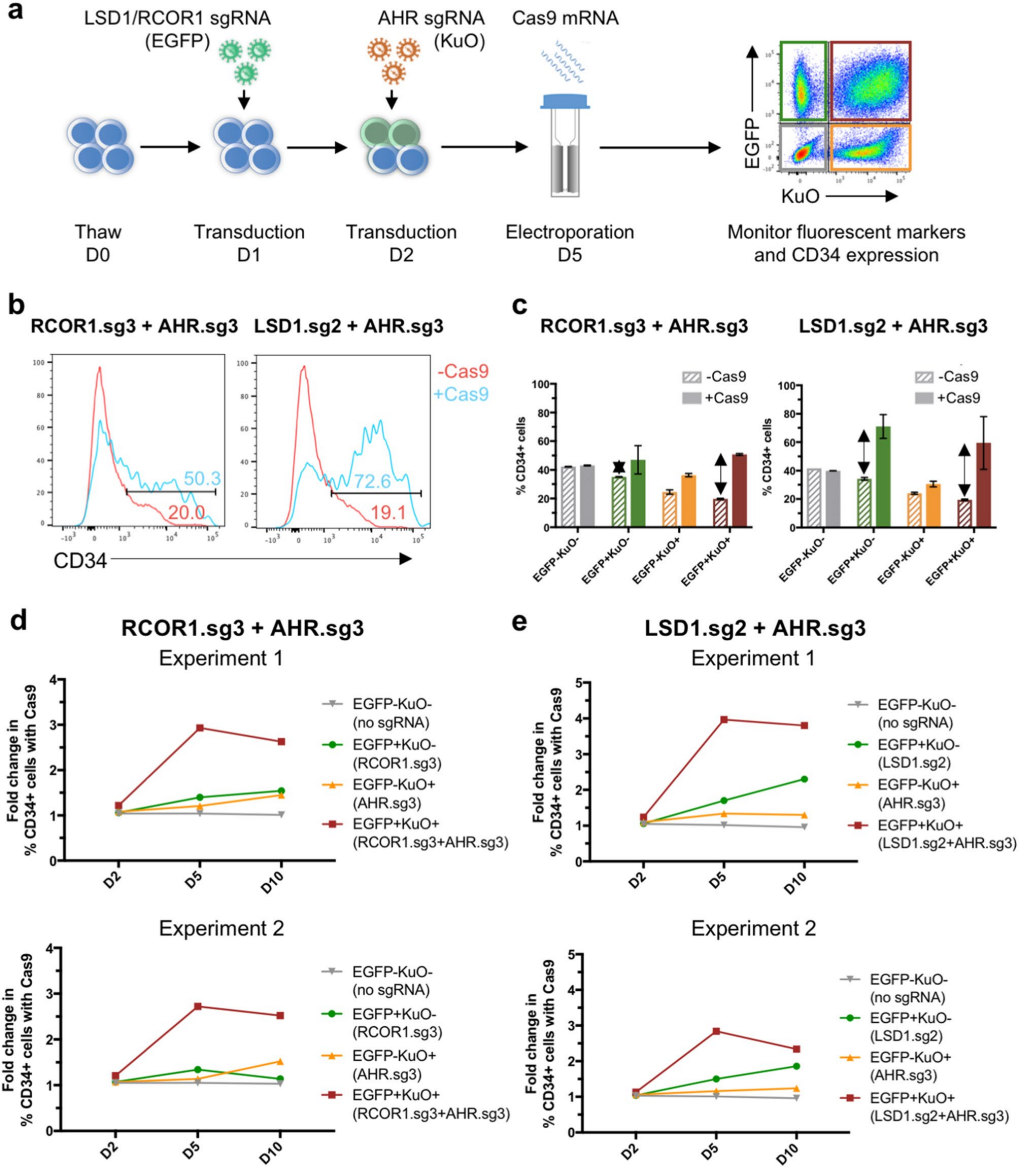
Combinatorial targeting of CoREST and AHR to propagate HSPCs ex vivo. (**a**) Schematic overview of experimental outline targeting LSD1 or RCOR1 and AHR. Thawed CD34^+^ cells were sequentially transduced on day 1 and day 2 with lentiviral sgRNAs targeting LSD1 or RCOR1 and AHR, containing EGFP or KuO, respectively. Cells were electroporated with Cas9 mRNA after 3 days in culture. Fluorescent marker and CD34 expression were monitored using flow cytometry. (**b**) Representative histograms showing the frequency of CD34^+^ cells in the double transduced (EGFP^+^KuO^+^) population without (− Cas9) or with electroporation of Cas9 mRNA (+ Cas9) for sgRNA combinations RCOR1.sg3 and AHR.sg3, and LSD1.sg2 and AHR.sg3 at day 10 post-electroporation. (**c**) Frequency of CD34^+^ cells in the transduced populations using sgRNA combinations RCOR1.sg3 and AHR.sg3, and LSD1.sg2 and AHR.sg3, without (− Cas9) or with electroporation of Cas9 mRNA (+ Cas9) at day 10 post-electroporation. (**d**,**e**) The fold change in frequency of CD34^+^ cells with electroporation of Cas9 mRNA compared to only transduced cells (− Cas9) for sgRNA combinations RCOR1.sg3 and AHR.sg3, and LSD1.sg2 and AHR.sg3 at day 2, 5, and 10 (D2–10) post-electroporation in two independent experiments.

We transduced CD34^+^ HSPCs with validated sgRNAs targeting LSD1, sg2 (Supplementary Fig. S3), or RCOR1, sg3^32^, harbouring EGFP, followed by transduction of lentiviral sgRNA targeting AHR, sg3^30^, containing KuO, followed by electroporation with Cas9 mRNA (Fig. 3a). CD34 expression within each of the transduced populations was monitored using flow cytometry at day 2, 5 and 10 post-electroporation. As a potentially confounding factor, we observed that the maintenance of CD34 expression differed somewhat between the transduced populations even when they had not been transfected with Cas9 (Supplementary Fig. S3). This is likely due to different transduction propensities for sub fractions of HSPCs, where quiescent cells are more resistant to transduction and cycling cells are more easily transduced^19,20^. Indeed, the non-transduced (EGFP^−^KuO^−^) population preserved CD34 expression as well as the combined expression of CD34 and CD90 better than the transduced populations. Moreover, the single transduced populations (EGFP^+^KuO^−^, EGFP^-^KuO^+^) maintained the expression better than double transduced cells (EGFP^+^KuO^+^) (Supplementary Fig. S3). Therefore, rather than directly comparing CD34 levels between the transduced populations, we assessed the relative ratio of CD34^+^ cells within each transduced population with or without Cas9 treatment. We found that cells that were double transduced (EGFP^+^KuO^+^) with either of the two sgRNA combinations (RCOR1.sg3 and AHR.sg3 or LSD1.sg2 and AHR.sg3) and electroporated with Cas9 mRNA, showed a profound increase in frequency of CD34^+^ cells at day 10 post-electroporation compared to double transduced cells without Cas9 (Fig. 3b,c), indicating a strong effect of promoting maintenance of CD34^+^ HSPCs. The sgRNA combination LSD1.sg2 and AHR.sg3 further displayed an increase in frequency of the more immature CD34^+^CD90^+^ cells at day 10 post-electroporation (Supplementary Fig. S3). This relative increase in CD34^+^ and CD34^+^CD90^+^ cells was not seen for non-transduced cells electroporated with Cas9 mRNA (Fig. 3c, Supplementary Fig. S3). Moreover, the relative increase in frequency of CD34^+^ cells in double transduced cells electroporated with Cas9 mRNA exceeded that of the two single transduced populations, suggesting a potential synergy or additive effect from targeting AHR in combination with components of the CoREST complex (LSD1 or RCOR1) (Fig. 3c–e). Targeting LSD1 alone showed a stronger effect on promoting maintenance of CD34^+^ cells compared to single transduced cells targeted for either RCOR1 or AHR (Fig. 3c–e).

To summarize, we used our split CRISPR/Cas9 delivery system to target AHR together with LSD1 or RCOR1 as gene targets that are known to expand CD34^+^ cells upon individual gene deletion. The potential synergistic effects from targeting AHR and LSD1 or AHR and RCOR1 requires further investigation but the experiments illustrate the feasibility of our double knockout system to identify possible co-operative events in primary human HSPCs.

## Discussion

Here, we report on the use of a split CRISPR/Cas9 delivery system for traceable double gene editing in primary human HSPCs. The use of lentiviral sgRNAs coupled to fluorescent markers on separate vectors enables tracing of single and double transduced cells and the editing within these populations, providing internal controls. We show that this system is efficient for editing two loci simultaneously in CB-derived CD34^+^ cells. Furthermore, we demonstrate the feasibility of the approach to investigate combinatorial effects and to model synthetic lethal interactions in human HSPCs.

We first attempted to achieve double editing using single bicistronic sgRNA vectors. In our hands, these one vector systems failed to express sufficient levels of sgRNAs for efficient editing in primary HSPCs, neither when using dual pol III promoters or small tRNA promoters to drive sgRNAs in tandem. Particularly, the use of two pol III promoter cassettes was problematic as we failed to detect substantial editing for neither of the genes targeted, despite their respective sgRNAs having shown highly efficient knockout when expressed from single sgRNA vectors. Further experiments would be required to understand the underlying mechanisms for this, but it is likely that the presence of two pol III promoters causes an interference with sgRNA expression from each of the cassettes^37^. The tRNA system was somewhat more efficient, yet we could not achieve the levels of editing that have been reported in cell line systems. Thus, there may be specific challenges in expressing multiple sgRNAs using one vector systems in primary cells such as HSCs. Furthermore, using standard protocols for the well-established RNP delivery method was relatively inefficient in terms of double-edited cells, and since this approach lacks a traceable component, the edited cells cannot be distinguished from non-edited cells. There are likely opportunities for optimization and multiple aspects of such systems that could be investigated further, but we chose not to proceed further with that within the current project. For example, it has been suggested that sequential delivery of RNP complexes could improve the efficiency of double editing^38^.

Our strategy to deliver the sgRNA components separately by lentiviral transduction including different fluorescent markers is advantageous in the sense that the individual transduced populations can be tracked using fluorescent-activated cell sorting, thereby allowing tracing of the respective editing events. This approach allows for editing of multiple genes of interest and their relationship to be investigated within a single system, providing internal controls in terms of specifically labelled single-edited populations and non-edited cells. However, efficient delivery of Cas9, together with optimal guide performance, is required since cells can only be traced by the expression of single or multiple sgRNAs, and not based on actual editing events. Here, Cas9 was delivered successfully to 70–80% of the transduced cells, which is reflected in the frequency of edited cells (Fig. 1C). To minimize incomplete gene editing, validated sgRNA sequences should be used in combination with an optimized Cas9 delivery system. In our previous work, we showed that the use of a chimeric backbone structure for sgRNAs greatly enhanced editing efficiency^14^ and we successfully applied that here as well. From the targeting of the cell surface markers CD45 and CD44, we could show that the vast majority of cells in each of the transduced EGFP and KuO subpopulations showed the expected editing event(s) (Fig. 1c,d), which should allow for meaningful functional tracing experiments. We conceptually demonstrated this by targeting STAG1 and STAG2, two paralog genes where a disruption in one gene is tolerated while disruption of both is lethal to the cell. Indeed, we saw a rapid decline of double transduced, edited cells, which was not seen for the single transduced, edited cells or non-transduced, non-edited cells. Furthermore, we observed an increase in CD34^+^ HSPCs in the single STAG2 edited population, consistent with previous reports that knockdown of STAG2 promotes maintenance of HSPCs^29,39^.

To test our system in terms of investigating combinatorial and potentially synergistic effects, we chose to target genes identified as ex vivo regulators of hematopoiesis, which is of relevance to cord blood expansion *in vitro*^40^. Currently, the AHR antagonist SR1 and the pyrimidoindole derivative UM171 are independently assessed in clinical trials for HSPC expansion^34–36^. We recently showed that UM171 mediates its expansion effects by targeting the CoREST complex for degradation^32^. Therefore, we decided to target the two CoREST members LSD1 and RCOR1, in combination with AHR using our double gene editing system.

During the experiments, we observed that the maintenance of CD34, as well as the combined CD34 and CD90 expression, differed between the transduced populations even without Cas9 treatment (Supplementary Fig. S3). The CD34^+^ population is heterogeneous and the sub fractions of HSPCs have different transduction propensities where quiescent cells are more resistant to transduction while cycling cells are more easily transduced^19,20^, which we also observed with regards to the double transduction (Supplementary Fig. S1). The unequal transduction efficiencies of CD34^+^ HSPCs make the direct comparison between the single-, double- and untransduced cell populations problematic especially when evaluating stemness properties. To avoid this caveat, it is important to make comparisons to sgRNA transduced cells without Cas9 treatment, as we applied in these experiments. Another limitation of our system is that it involves quite extensive manipulation of the cells over several days with sequential sgRNA transductions followed by Cas9 electroporation. Especially the electroporation procedure induces significant cell death and the extended time in culture will inevitably trigger differentiation and partial loss of HSPCs.

From our proof of concept-experiments, we saw enhanced frequencies of CD34^+^ cells in the single-edited populations, which was augmented when targeting both genes for all sgRNA combinations tested. This indicates a combined and potentially synergistic effect when simultaneously targeting AHR and CoREST. Previously, Fares et al. have reported on the cooperation of the small molecules SR1 and UM171 (targeting AHR and CoREST, respectively) to enhance ex vivo expansion of progenitor cells^41^. Our findings provide genetic support to this observation. Further investigation of the potential synergistic effect from targeting these pathways could potentially highlight new mechanisms and optimization opportunities for HSPC ex vivo expansion. A genetic model provides a more precise tool for such studies compared to small molecules that are likely to have a broader range of off-target effects.

In conclusion, we have developed a traceable CRISPR/Cas9 system that enables multiplexed editing in human HSPCs for studies of gene dependencies and gene interactions. The system will serve as a valuable tool for modelling polygenic diseases in primary HSPCs and to elucidate underlying cellular functions of gene interactions in these clinically relevant cells.

## Methods

### Cloning

To add a second promotor to the modified version of the pLentiCRISPR v.2 vector (Addgene vector #52,961, denoted pLCv2)^14^, the H1 promotor^16^ and a second sgRNA with chRNA2 backbone was inserted after the U6-sgRNA cassette through restriction enzyme cloning. For cloning of tRNA sequences into the pLCv2 vector, the U6 promoter was amplified and cloned in reverse orientation followed by tRNA sequences Gln and Pro^18^ with selected sgRNAs together with the chRNA2 backbone, also in reverse orientation, using restriction enzyme cloning. For the use of two separate sgRNA vectors for multiplexed editing, sgRNA sequences were cloned into the pLCv2 vector using BsmBI sites as previously described^14^. The EGFP marker was exchanged for KuO using restriction enzyme cloning. All sgRNA sequences can be found in Supplementary Table 1.

### Primary human samples

Umbilical cord blood samples were collected at the maternity wards of Helsingborg General Hospital and Skåne University Hospital in Lund and Malmö, Sweden. The samples were collected after informed, written consent with approval from the regional ethical committee (Regionala Etikprövningsnämnden i Lund/Malmö, approval #2010–696). All experiments were performed in accordance with these guidelines and regulations. Density-gradient centrifugation (Lymphoprep, Abbott Rapid Diagnostics) was used to separate the mononuclear cell fraction followed by magnetic bead purification (Miltenyi Biotec) to isolate CD34^+^ HSPCs, which were stored in − 80 °C until use.

### In vitro culture

Cas9 was expressed in HL60 cells, kindly provided by Associate Prof. Mattias Magnusson, through transduction of a modified version of the pLCv2 vector containing a Cas9-P2A-BFP cassette. Transduced cells were then selected based on fluorescent marker expression. The Cas9-expressing HL60 cells were cultured in HyClone RPMI-1640 Medium (Cytiva) supplemented with 10% heat-inactivated FBS (Cytiva) and 1% Penicillin–Streptomycin (Cytiva). Primary CD34^+^ HSPCs were thawed and cultured in Serum-Free Expansion Medium (SFEM, Stem Cell Technologies) complemented with 1% Penicillin–Streptomycin (Cytiva) and Stem cell factor (SCF), FLT3-ligand (FLT3L) and thrombopoietin (TPO) were added in the concentration 100 ng/ml and acquired from PeproTech or Miltenyi Biotec. The CD34^+^ cells were cultured at 37 °C, 5% CO_2_ for 24 h prior to lentiviral transduction and 48 h prior to RNP electroporation.

### Lentiviral production

Lentiviruses harbouring sgRNA(s) and fluorescent marker were produced in the human embryonic kidney (HEK) 293 T cell line (DSMZ, German Collection of Microorganisms and Cell Cultures). HEK293T cells were co-transfected with a mix of transfer plasmid, packaging constructs, VSV-G-encoding envelope and Polyethylenimine (Polysciences). Viral supernatants were harvested after 48 and 72 h, then filtered (0.45 µm) and concentrated 100-fold by ultracentrifugation (Beckman Coulter) before storage in − 80 °C until use.

### Lentiviral transduction

Lentivirus was thawed on ice and added to 50 000 cells in 200 µl culture medium in a 96 well plate. Cells were transduced at a multiplicity of infection (MOI) of 10, with a target transduction efficiency of 40–50%. For sequential double transduction, cells were transduced at MOI 10 at day 1 post-thaw, followed by a half a medium change on day 2 before transduction with the second lentiviral sgRNA vector at MOI 20 based on the starting cell numbers to compensate for the approximate doubling in cell numbers that occurred between day 1 and day 2. The target transduction efficiency for these transductions were 100%. Cells were cultured at 37 °C, 5% CO_2_ for 72 h prior to electroporation.

### Electroporation of CB CD34^+^ hematopoietic stem and progenitor cells

CD34^+^ HSPCs were electroporated as described previously^14^. In brief, Cleancap Cas9 mRNA (TriLink BioTechnologies) was thawed and incubated on ice until electroporation. For electroporation with RNPs, 3 µg Cas9 protein (PNA Bio) in storage buffer and synthetic sgRNA (Synthego) in storage buffer (molar ratio 1:2.5) were incubated for 10 min at room temperature. For targeting two loci, RNPs for each target gene were incubated separately and then mixed prior to electroporation. 100 k transduced or untreated CD34^+^ cells were resuspended in BTXpress Electroporation Solution (Harvard Apparatus) and added to 2–3 µg Cas9 mRNA or Cas9:sgRNA RNPs, respectively, to a total volume of 20 µl, which was then transferred to 1 mm cuvettes (Harvard Apparatus). Cells were immediately electroporated using the BTX ECM 830 Electroporation System (Harvard Apparatus) with the following parameters set: 125 V, 950 µF, 5 ms, Ω none, Mode LV. Cells were recovered for 10 min at room temperature prior to culture at 37 °C, 5% CO_2_.

### Flow cytometry

CD34^+^ HSPCs were stained with CD45-AlexaFluor700 (BioLegend, clone H130), CD34-PE/Cy7 (BioLegend, clone 581) and CD44-BV421 (BioLegend, clone IM7). All antibodies were used in the concentration recommended by the manufacturer. Debris and dead cells were omitted by forward scatter (FSC), side scatter (SSC), DAPI or 7-AAD staining (1:100 dilution prior to analysis). The flow cytometry analyses were performed using the BD LSRFortessa instrument (BD Biosciences) and cells were sorted using BD FACSAria IIu (BD Biosciences). The software FlowJo (FlowJo, LLC) was used to analyse the data.

### Western blot

Cas9-expressing HL60 cells were transduced with lentivirus containing EGFP or KuO and sgRNA targeting STAG1, STAG2 or LSD1, aiming at a transduction efficiency of 100%. Cells were harvested on day 4 or 5 post-transduction and washed 3 times with phosphate-buffered saline and then collected in RIPA Lysis and Extraction Buffer (Thermo Fisher Scientific) supplemented with protease inhibitor cocktail (Thermo Fisher Scientific). Further, samples were mixed with Laemmli buffer (Bio-Rad), supplemented with β-mercaptoethanol (Sigma-Aldrich) and protease inhibitor cocktail (Thermo Fisher Scientific), and denatured at 95 °C for 2–5 min. Western blotting was performed using the NuPAGE electrophoresis system (Thermo Fisher Scientific) according to the manufacturer’s instructions using MagicMark XP Western Protein Standard (Invitrogen) and Spectra Multicolor Broad Range Protein Ladder (Thermo Fisher Scientific). After protein transfer using iBlot 2 Gel Transfer Device (Thermo Fisher Scientific) according to the manufacturer’s instructions, the membrane was cut in two parts, which contained high molecular weight and low molecular weight proteins, respectively. The delineation of membranes was based on the well-known molecular weight of investigated proteins. The membrane parts were separately incubated with the corresponding primary antibody. The following primary antibodies were used: mouse anti-actin ab-5 mAb clone C4/actin (BD Biosciences), rabbit anti-SA1 C-terminal pAb (dilution 1:X, Abcam), rabbit anti-STAG2 pAb (dilution 1:X, Cell Signaling Technology) and rabbit anti-LSD1 mAb clone C69G12 (Cell Signaling Technology). Primary antibodies were used at dilution 1:1000 except for mouse anti-actin, which was used at dilution 1:10 000. After washing 3 times with PBS-T (Thermo Fisher Scientific), the membranes were incubated with the appropriate secondary antibody, Amersham ECL horseradish peroxidase-conjugated F(ab’)_2_ fragment of sheep anti-mouse IgG at dilution 1:10 000 (Cytiva) or donkey anti-rabbit IgG at dilution 1:5000 (Cytiva). The membranes were incubated with Amersham ECL Select Western Blotting Detection Reagent (Cytiva) and proteins were visualized with ChemiDoc XRS + System (Bio-Rad).

### Statistical analysis

GraphPad Prism 7 (GraphPad Software) was used to perform One-Way analysis of variance (ANOVA) with Tukey’s multiple comparisons test to compare multiple groups. Error bars represent standard deviation (S.D.) unless indicated otherwise and significance is indicated with asterisks (**** p < 0.0001).

## Supplementary Information

Supplementary Information.

## Data Availability

The datasets generated during and/or analysed during the current study are available from the corresponding author on reasonable request.
