# Supplementary material for: Combinatorial gene targeting in primary human hematopoietic stem and progenitor cells
Source: Sci Rep. 2022 Oct 28;12:18169. doi: 10.1038/s41598-022-23118-8 (PMC9616885; doi:10.1038/s41598-022-23118-8)
Supplement: Supplementary file 1 — Supplementary Information. [file 41598_2022_23118_MOESM1_ESM.pdf]

Supplementary Information

Combinatorial Gene Targeting in Primary Human Hematopoietic Stem and Progenitor Cells

Alexandra Bäckström[1], David Yudovich[1], Kristijonas Žemaitis[1], Ludvig Nilsén Falck[1], Agatheeswaran Subramaniam[1] and Jonas Larsson[1*]

[1]Division of Molecular Medicine and Gene Therapy, Lund Stem Cell Center, Lund University, Lund, Sweden

*Correspondence to Jonas Larsson MD PhD, Molecular Medicine and Gene Therapy, BMC A12, 221 84, Lund, Sweden; phone: +46-46-2220580; fax: +46-46-2220568;
e-mail: jonas.larsson@med.lu.se

Supplementary Table 1. sgRNA sequences

Gene target	Guide	Sequence
AHR	sg3	CTTACTTCGGATATGGGACT
CD44	sg4	CCAGCTATTGTTAACCGTGA
CD45	sg8	GGGCGCACAGGAACCTATAT
LSD1	sg1	TGTGGTCCACTGATAATATC
LSD1	sg2	AGAGCCGACTTCCTCATGAC
Non-targeting	ntB	TACTAACGCCGCTCCTACAG
Non-targeting	ntC	TGAGGATCATGTCGAGCGCC
RCOR1	sg3	GCGGAGAGGGAGGAACAACG
STAG1	sg1	TGGACACCCTCAACAGAATG
STAG1	sg2	ACTACTTCAGAAACGCAAAG
STAG1	sg3	TGGCTGGACTCTTCATGACA
STAG2	sg1	TGAGCATGCAGCATACCTTG
STAG2	sg2	AGTCCCACATGCTATCCACA

Note: The sgRNA sequences not starting with a 5’G were cloned into the lentiviral vectors with an additional 5’G.

Supplementary Figure S1

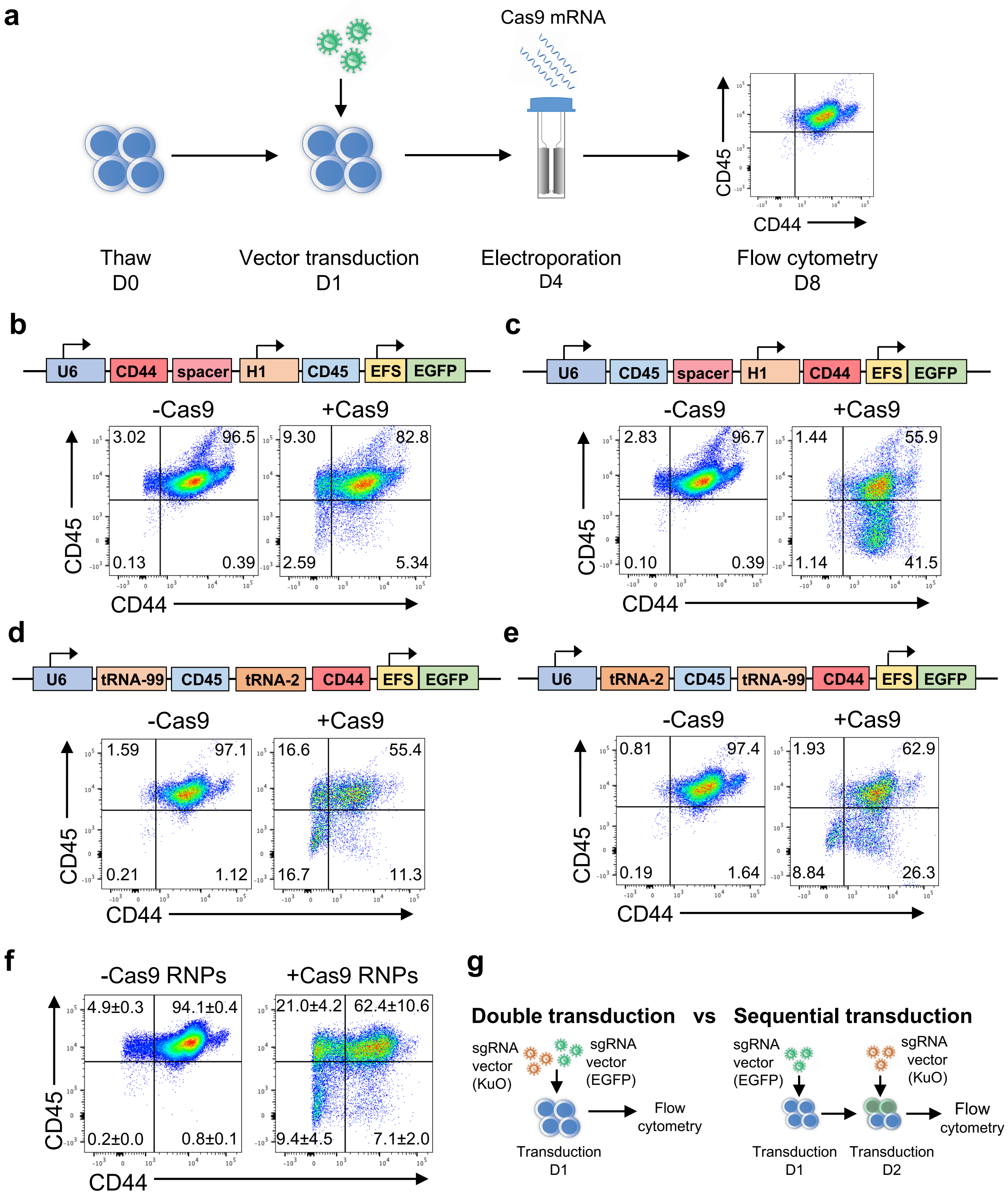

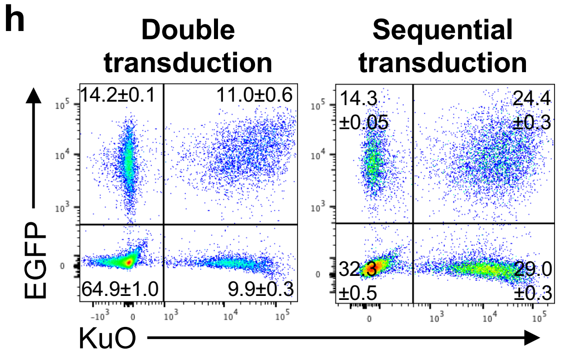

i

Sub fraction	Frequency of double transduced (EGFP+KuO+) cells (%)
CD34low	25.2±0.5
CD34mid	25.9±0.5
CD34high	15.4±0.5

Supplementary Figure S1. Double gene editing in $CD34^+$ HSPCs using bicistronic sgRNA vectors

(a) Schematic of experimental outline using bicistronic sgRNA vectors. Thawed $CD34^+$ cells were transduced with lentiviral sgRNA vector targeting CD44 (sg4) and CD45 (sg8) containing EGFP on day 1. Cells were electroporated with Cas9 mRNA at day 4. The transduction efficiency and editing were assessed using flow cytometry 4 days post-electroporation on day 8 (n=2). **(b and c)** $CD34^+$ HSPCs were transduced with lentivirus containing two promoter-sgRNA cassettes as indicated and electroporated with Cas9 mRNA. The representative FACS plots show CD45 and CD44 editing in $CD34^+EGFP^+$ cells without (-Cas9) or with electroporation of Cas9 mRNA (+Cas9) on day 8. **(d and e)** $CD34^+$ HSPCs were transduced with lentivirus containing two tRNA-sgRNA cassettes as indicated and electroporated with Cas9 mRNA. The representative FACS plots show CD45 and CD44 editing in $CD34^+EGFP^+$ cells without (-Cas9) or with electroporation of Cas9 mRNA (+Cas9) on day 8. **(f)** Representative FACS plots showing CD45 and CD44 editing in $CD34^+$ cells without (-Cas9 RNPs) or with electroporation of Cas9:sgRNA RNPs (+Cas9 RNPs) at 4 days post-electroporation (n=3). **(g)** Schematic overview of double and sequential vector transduction. $CD34^+$ cells were transduced with both lentiviral sgRNA vectors on day 1 or sequentially transduced with lentiviral sgRNA vector containing EGFP on day 1 and lentiviral sgRNA vector containing KuO on day 2. Transduction efficiencies were assessed using flow cytometry. **(h)** Representative examples of FACS plots showing EGFP and KuO expression in $CD34^+$ cells following double (n=2) or sequential transduction (n=3). **(i)** Table showing the frequency of double transduced (EGFP+KuO+) cells (%) for sub fractions of CD34+ cells (n=3).

Supplementary Figure S2

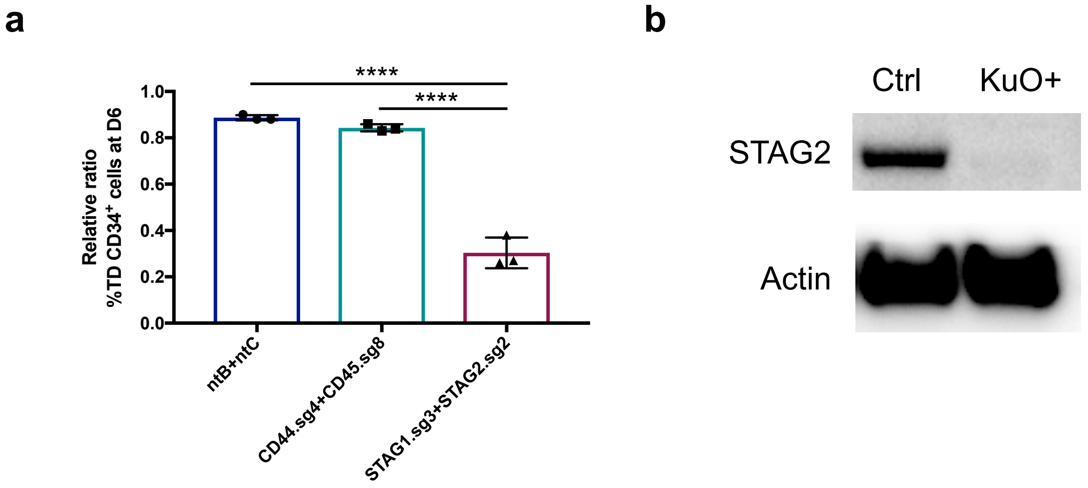

Supplementary Figure S2. Modelling the synthetic lethality of STAG1 and STAG2 in CD34$^+$ HSPCs

(a) Relative ratio of the frequency of transduced (TD) CD34$^+$ cells at day 6 compared to day 2 post-electroporation for cells transduced with guide combination non-targeting B and C (ntB+ntC), CD44 and CD45 (CD44.sg4+CD45.sg8), or STAG1 and STAG2 (STAG1.sg3+STAG2.sg2). Statistical significance was calculated using One-Way ANOVA with Tukey's multiple comparisons test. ****$p < 0.0001$. **(b)** Western blot analysis of STAG2 protein in untreated (Ctrl) and sorted EGFP$^-$KuO$^+$ (KuO+) cells at day 13 post-electroporation. STAG2 and Actin bands were cropped from different parts of the same blot. Note that the actin part of the membrane is overexposed. However, this part of the blot shows that the actin protein is present in all samples. This blot has not been used for quantification. Original blots are shown in Supplementary Figure S4.

Supplementary Figure S3

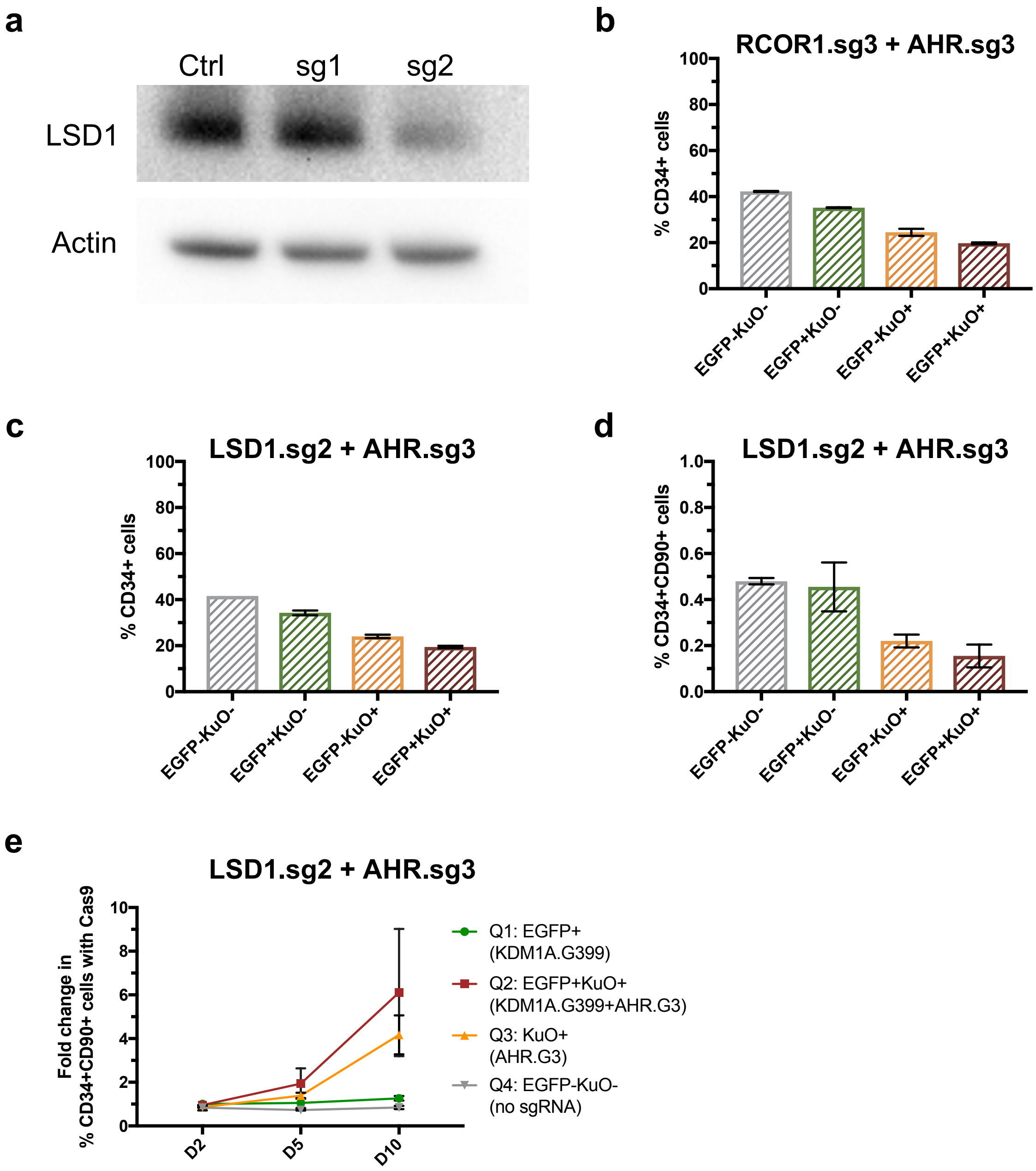

Supplementary Figure S3. Combinatorial targeting of CoREST and AHR to propagate HSPCs *ex vivo*

(a) Western blot analysis of LSD1 protein in non-transduced (Ctrl) and sgRNA transduced (sg1, sg2) Cas9 expressing-HL60 cells. LSD1 and Actin bands were cropped from different parts of the same blot. Original blots are shown in Supplementary Figure S4. **(b and c)** Frequency of $CD34^{+}$ cells in the transduced populations for sgRNA combinations RCOR1.sg3 and AHR.G3, and LSD1.sg2 and AHR.G3 without electroporation of Cas9 mRNA (-Cas9) at day 10 post-electroporation. **(d)** Frequency of $CD34^{+}CD90^{+}$ cells in the transduced populations for sgRNA combination LSD1.sg2 and AHR.sg3 without electroporation of Cas9 mRNA (-Cas9) at day 10 post-electroporation. **(e)** The fold change in frequency of $CD34^{+}CD90^{+}$ cells with electroporation of Cas9 mRNA compared to only transduced cells (-Cas9) for sgRNA combination LSD1.sg2 and AHR.sg3 at day 2, 5, and 10 (D2-10) post-electroporation.

Supplementary Figure S4

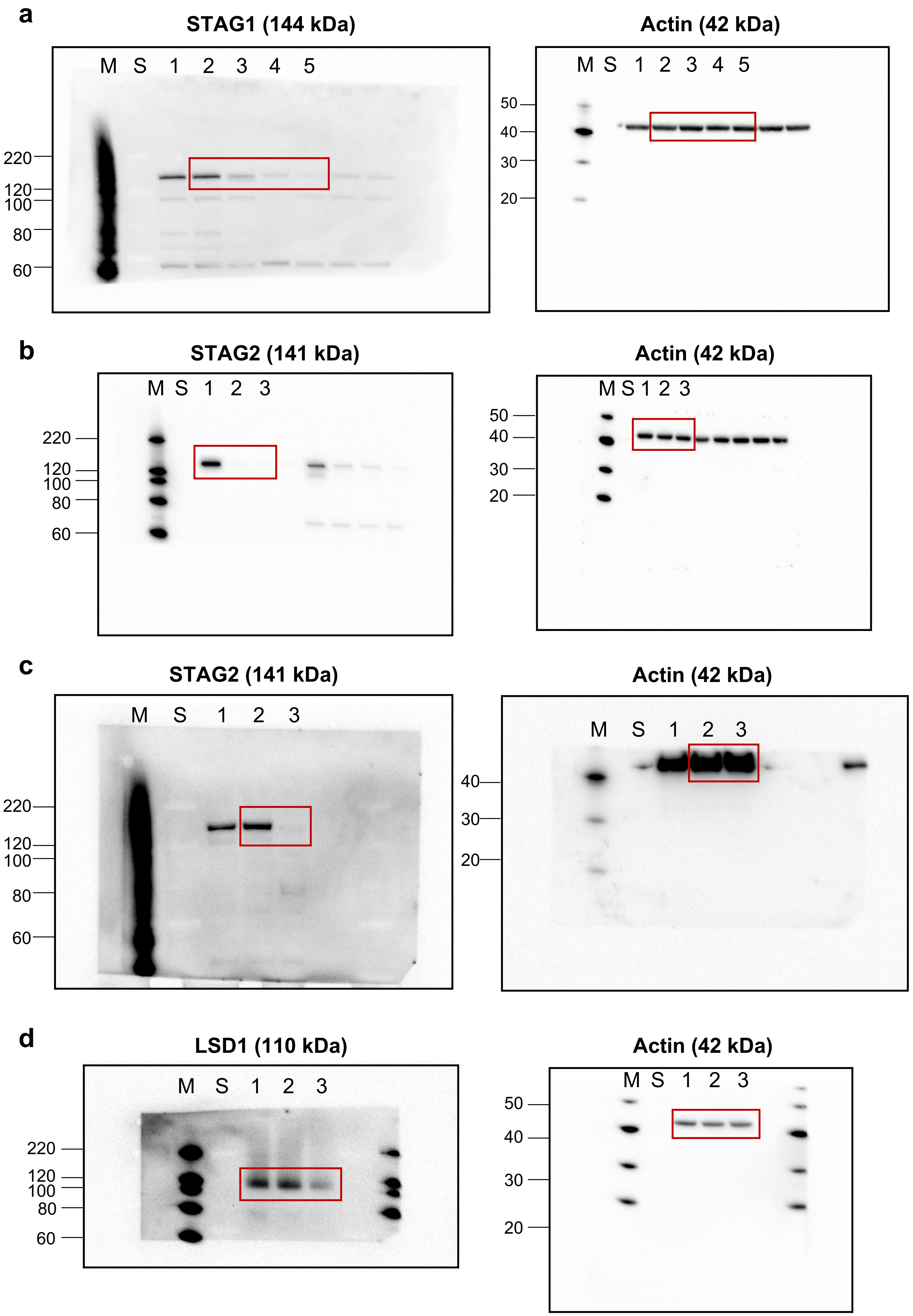

Supplementary Figure S4. Full size blot images used in Figure 2, S2, and S3. MagicMark XP Western Protein Standard (M) and Spectra Multicolor Broad Range Protein Ladder (S) were used as protein molecular weight references. Membranes were cut in two parts, which contained high molecular weight and low molecular weight proteins, respectively. The membrane parts were separately incubated with the corresponding primary and secondary antibodies. For more details, see method section. Squares in red solid lines show cropped parts. **(a)** Original blot Figure 2b. STAG1 and actin blot: 1. Untreated (Ctrl) 2. Untreated (Ctrl) 3. sg1 4. sg2 5. sg3. **(b)** Original blot Figure 2b. STAG2 and actin blot: 1. Untreated (Ctrl) 2. sg1 3. sg2. **(c)** Original blot Supplementary Figure S2. STAG2 and actin blot: 1. Untreated cells (Ctrl) 2. Untreated cells (Ctrl) 3. KuO^{+} cells. Note that the actin part of the membrane is overexposed. However, this part of the blot shows that the actin protein is present in all samples. This blot has not been used for quantification. **(d)** Original blot Supplementary Figure S3. LSD1 and actin blot: 1. Untreated (Ctrl) 2. sg1 3. sg2.
